# Treatment access gap during the COVID-19 Pandemic: impact on problematic alcohol use and the moderating roles of perceived stress and resilience

**DOI:** 10.3389/fpsyt.2024.1487277

**Published:** 2024-12-12

**Authors:** Rhianna R. Vergeer, Jeremy W. Luk, Bethany L. Stangl, Emma M. McCabe, Ugne Ziausyte, Melanie L. Schwandt, David Goldman, Vijay A. Ramchandani, Nancy Diazgranados

**Affiliations:** ^1^ Human Psychopharmacology Laboratory, Division of Intramural Clinical and Biological Research (DICBR), National Institute on Alcohol Abuse and Alcoholism (NIAAA), Bethesda, MD, United States; ^2^ Office of the Clinical Director, DICBR, NIAAA, Bethesda, MD, United States

**Keywords:** alcohol use, resilience, stress, COVID-19, access, barriers, treatment utilization, psychological treatment

## Abstract

**Objective:**

The COVID-19 pandemic may have interfered with individuals’ access to alcohol use disorder (AUD) treatment, but limited research has documented the impact of treatment interference on drinking behavior. This study’s purpose was to examine the associations of AUD treatment interference with problematic alcohol use, and the moderating roles of perceived stress and resilience.

**Method:**

A cross-sectional survey design was employed. Data were drawn from the baseline assessment of the National Institute on Alcohol Abuse and Alcoholism COVID-19 Pandemic Impact on Alcohol Study. Between June 2020 and March 2021, 288 participants (48.6% female, 51.4% male) responded to key measures of interest by phone and/or through an online survey. Study hypotheses were tested using multiple linear regression models adjusted for demographic characteristics (age, sex, race, ethnicity, years of education, household income, marital status), study enrollment phase, and history of AUD.

**Results:**

Self-reported AUD treatment interference was positively associated with problematic alcohol use as measured by the Alcohol Use Disorders Identification Test (*b* = 2.05, *p* < 0.001). Significant moderation effects indicated the association between AUD treatment interference and problematic alcohol use was stronger at a high level of perceived stress (*b* = 3.08, *p* < 0.001) and was attenuated at a high level of resilience (*b* = -0.13, *p* = 0.874).

**Conclusions:**

Self-reported AUD treatment interference may indicate interruption to individuals’ support systems and highlight the need for continued access to treatment. Fostering positive coping strategies and resilience may help individuals mitigate risks of problematic drinking amidst a public health crisis.

## Introduction

The World Health Organization (WHO) estimated that one third to one half of mental health and substance use services were disrupted during the COVID-19 pandemic (1). Alcohol use disorder (AUD) treatment was among the most impacted services (1). In medical and hospital settings, addiction treatment was reduced partially due to furloughed staff, reduced hours, and redeployment of primary care and emergency providers to manage the swell of COVID-19 patients (2). Moreover, public health regulations that banned or discouraged in-person gatherings also prevented individuals from attending mutual-help groups that may be important to individuals in recovery (2). Given the temporary reduction in addiction treatment and service options (3), it is important to assess the extent that AUD treatment interference may be associated with problematic alcohol use during the COVID-19 pandemic.

The pandemic has posed challenges to traditional forms of addiction treatment. For example, some patients reported that therapy appointments were delayed or canceled and therapeutic relationships with their providers were harmed by pandemic-related stress (4, 5). In one study, 77% of adults in recovery from AUD felt the pandemic negatively impacted their recovery (6). To support patients, many addiction treatment programs and mutual-help groups have switched to providing services virtually (2, 4–6). Telemedicine can make addiction treatment more accessible (7–10), leading to increased patient attendance (2), decreased drop-out rates (11), and more flexibility to attend mutual-help groups (6). Yet, some patients may not find telemedicine to be as helpful, citing difficulties making personal connections, technological challenges, concentration problems, and a lack of a quiet environment to attend appointments (4, 6, 8, 11). Given these mixed reports, it is worthwhile to examine the potential impact of pandemic interference with AUD treatment on problematic alcohol use during the pandemic. Of note, a key literature gap in this area is the lack of exploration of moderating factors that may strengthen or weaken the association between treatment interference and problematic alcohol use.

Contextual influences and individual differences may moderate the association between AUD treatment interference and pandemic problematic alcohol use. Specifically, this association may be strengthened by high perceived stress, as stress is linked to maladaptive coping and problematic alcohol use, especially in the context of the pandemic (12–15). Conversely, resilience is a protective factor against problematic alcohol use (16–18) and thus may be a buffer against the adverse impact of AUD treatment interference. The identification of moderators of the AUD treatment interference and problematic alcohol use can help inform prevention and intervention efforts, such as the relevance of stress management and resilience training within addiction treatment (19).

In this study, we investigated the association between pandemic-related AUD treatment interference and problematic alcohol use and tested the moderating roles of perceived stress and resilience. We hypothesized that AUD treatment interference would be positively associated with problematic alcohol use. We further hypothesized that high perceived stress would strengthen the association between AUD treatment interference and problematic alcohol use, whereas high resilience would buffer against the adverse impact of AUD treatment interference on problematic alcohol use.

## Method

### Participants

We utilized baseline data from the National Institute on Alcohol Abuse and Alcoholism (NIAAA) COVID-19 Pandemic Impact on Alcohol (C19-PIA) Study. The C19-PIA study was approved by the National Institutes of Health Intramural Institutional Review Board and is registered in clinicaltrials.gov (NCT04391816). Each participant gave informed consent for participation in the C19-PIA study. Details about the sampling strategy and study procedures were reported previously (13, 20, 21).

**Figure 1:**
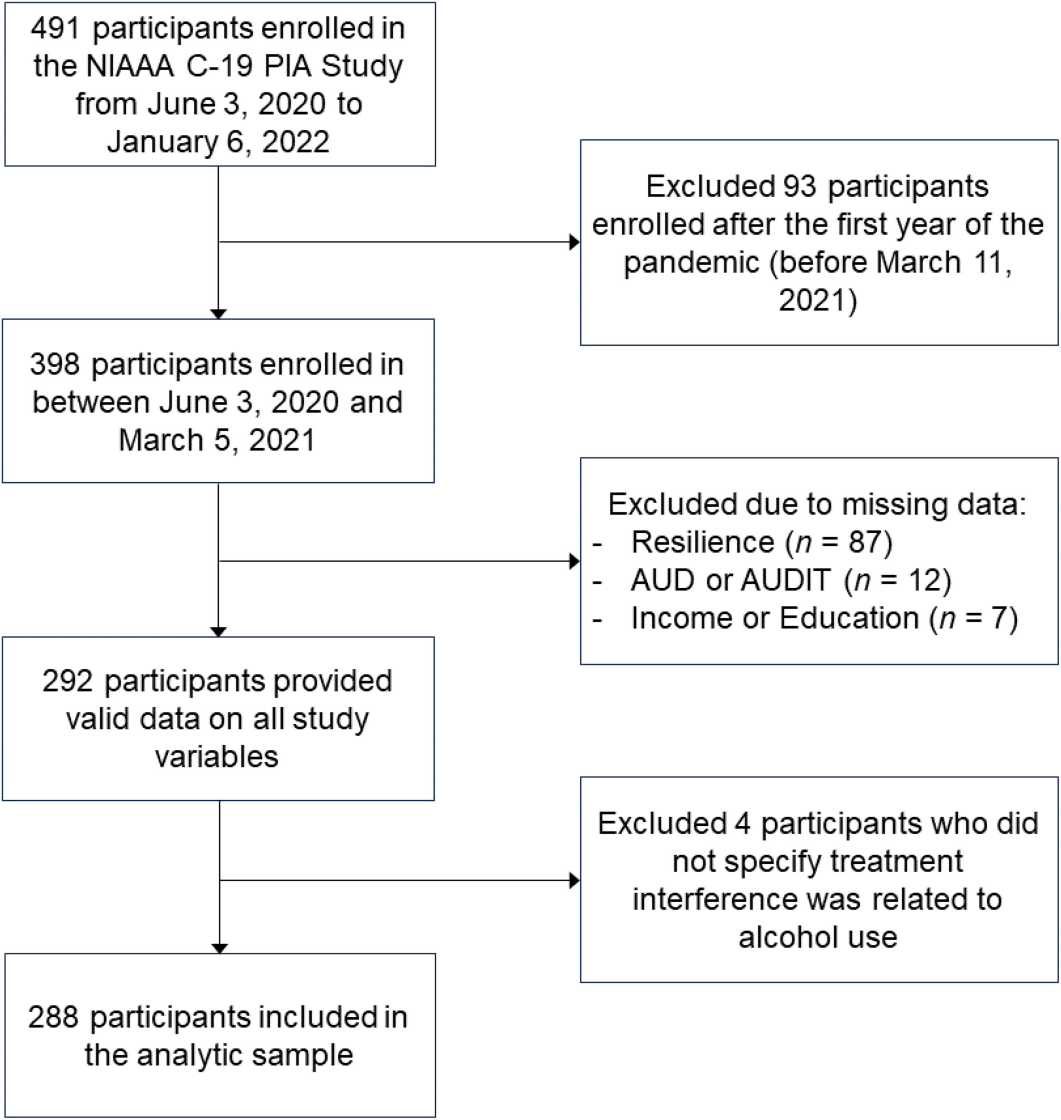
Flow diagram illustrating participant inclusion and exclusion in the current study.

The flow diagram presented in **Figure 1** illustrates the inclusion and exclusion of participants into the current analysis. First, as we conceptualized treatment interference to be most relevant during the first year of the COVID-19 pandemic, we excluded 93 participants who enrolled on or after March 11, 2021. Of the 398 participants who completed the C19-PIA baseline survey within the first year of the COVID-19 pandemic, 292 (73.4%) provided valid data on all study variables and were retained in the analytic sample. Four participants who did not specify the treatment interfered with by COVID-19 was related to alcohol use were excluded, yielding a final analytic sample of 288 adults. Most participants were current drinkers, and some met criteria for AUD prior to the pandemic. The history of AUD variable was assessed using the Structured Clinical Interview for DSM-IV or DSM-5 (22) as part of the NIAAA Natural History Protocol (NCT02231840), from which all participants were identified and subsequently contacted for participation in the C19-PIA Study. As the development of the pandemic did not follow a linear trend, the timing of enrollment into the C19-PIA Study was stratified into 3 phases while referencing infection statistics and local policies in the metro Washington DC area: June 3, 2020 to July 31, 2020 (Phase 1; 26.4%), August 1, 2020 to November 22, 2020 (Phase 2; 38.5%), and November 23, 2020 to March 3, 2021 (Phase 3; 35.1%). Enrollment phase was included in all adjusted analyses.

### Measures

#### AUD treatment interference

The AUD treatment interference survey item was embedded as part of a list of beliefs and experiences related to the COVID-19 pandemic. Thus, all participants were asked to rate their degree of agreement with the question: “Coronavirus has directly or indirectly prevented me from getting or interfered with treatment for my alcohol or substance use disorder.” Response options for the five-point Likert scale were 1 “strongly disagree”, 2 “disagree”, 3 “neutral”, 4 “agree”, and 5 “strongly agree”. The item was accompanied by an optional qualitative question asking the participant to specify the treatment interference experienced.

#### Problematic alcohol use

The 10-item Alcohol Use Disorders Identification Test (AUDIT; 23) is a widely used self-report measure developed by the WHO to screen for past year problematic drinking. Total AUDIT scores were computed (α = 0.93; possible range from 0-40), with a score of ≥8 indicating problematic alcohol use.

#### Perceived stress

The 10-item Perceived Stress Scale (PSS; 24) is a validated self-report questionnaire that assesses the frequency of stress experienced over the past month. Total PSS scores were computed (α = 0.91; possible range from 0-40), with higher scores indicating higher perceived stress.

#### Resilience

The 25-item Connor-Davidson Resilience Scale (CDRS; 25) is a self-report questionnaire of resiliency. Response options for the five-point Likert scale ranged from 0 “not true at all” to 4 “true nearly all the time”. Total CDRS scores were computed (α = 0.96; possible range from 0-100), with higher scores indicating higher resiliency.

### Analyses

Three multiple linear regression models were utilized to test the direct association between AUD treatment interference and problematic alcohol use (model 1) and conditional associations moderated by perceived stress (model 2) and resilience (model 3). Unadjusted analyses were first conducted. Next, we conducted adjusted analyses controlling for the following covariates: age, sex, race, ethnicity, years of education, household income, marital status, study enrollment phase, and history of AUD. For any significant interactions, simple slope analyses were conducted to decompose the interactions and the interactions would be visualized to aid interpretation. Analyses were conducted in Stata 17.0 (26). Sensitivity analyses were also conducted among current drinkers only (*n* = 237) after excluding 51 individuals with an AUDIT score of 0.

## Results

Descriptive statistics are presented in **Table 1**. Among participants, 30.6% (*n* = 88) “strongly disagreed” that the pandemic had interfered with their AUD treatment, 55.2% (*n* = 159) “disagreed”, 6.6% (*n* = 19) were “neutral”, 4.5% individuals (*n* = 13) “agreed”, and 3.1% “strongly agreed” (*n* = 9). Examples of how COVID-19 interfered with AUD treatment included Alcoholics Anonymous meetings being online and being unable to see one’s therapist or enter inpatient treatment.

**Table 1:** Descriptive statistics of study variables.

Variable	Full Sample (*N* = 288)
	*M* (*SD*)
*^a^* COVID-19 AUD Treatment Interference	1.9 (0.9)
Problematic Alcohol Use (AUDIT) (0-40)	7.1 (9.6)
Perceived Stress (PSS) (0-40)	14.5 (8.1)
Resilience (CDRS) (0-100)	67.9 (17.5)
Age (years)	44.2 (14.5)
Variable	% (*n*)
History of AUD	
Did not meet criteria for AUD	61.8 (178)
Met criteria for AUD	38.2 (110)
Sex	
Male	51.4 (148)
Female	48.6 (140)
Race	
White	53.8 (155)
Black/African American	30.2 (87)
Other *^b^*	16.0 (46)
Ethnicity	
Non-Hispanic	88.5 (255)
Hispanic or Latino/a/x	8.0 (23)
Unknown	3.5 (10)
Variable	Full Sample (*N* = 288)
	% (*n*)
Years of Education	
<13 years	20.5 (59)
13-16 years	54.9 (158)
≥17 years	24.7 (71)
Annual Household Income	
<$20,000	22.2 (64)
$20,000 – $74,999	45.8 (132)
≥$75,000	31.9 (92)
Marital Status	
Single	63.5 (183)
Married	22.2 (64)
Other *^c^*	14.2 (41)
Study Enrollment Phase	
Phase 1 (6/3/2020 – 7/31/2020)	26.4 (76)
Phase 2 (8/1/2020 – 11/22/2020)	38.5 (111)
Phase 3 (11/23/2020 – 3/03/2021)	35.1 (101)

AUD, alcohol use disorder; AUDIT, Alcohol Use Disorders Identification Test total score; PSS, Perceived Stress Scale; CDRS, Connor-Davidson Resilience Scale.

a COVID-19 AUD treatment interference was measured on a five-point Likert scale from 1 “strongly disagree” to 3 “neutral” to 5 “strongly agree”.

b “Other” race included the following: Asian (*n* = 25); American Indian/Alaska Native (*n* = 1); Multiracial (*n* = 7); and Unknown (*n* = 13).

c “Other” marital status included the following: divorced (*n* = 22); separated (*n* = 7); widowed (*n* = 5); other (*n* = 2); and “not provided” (*n* = 5).

**Figure 2:**
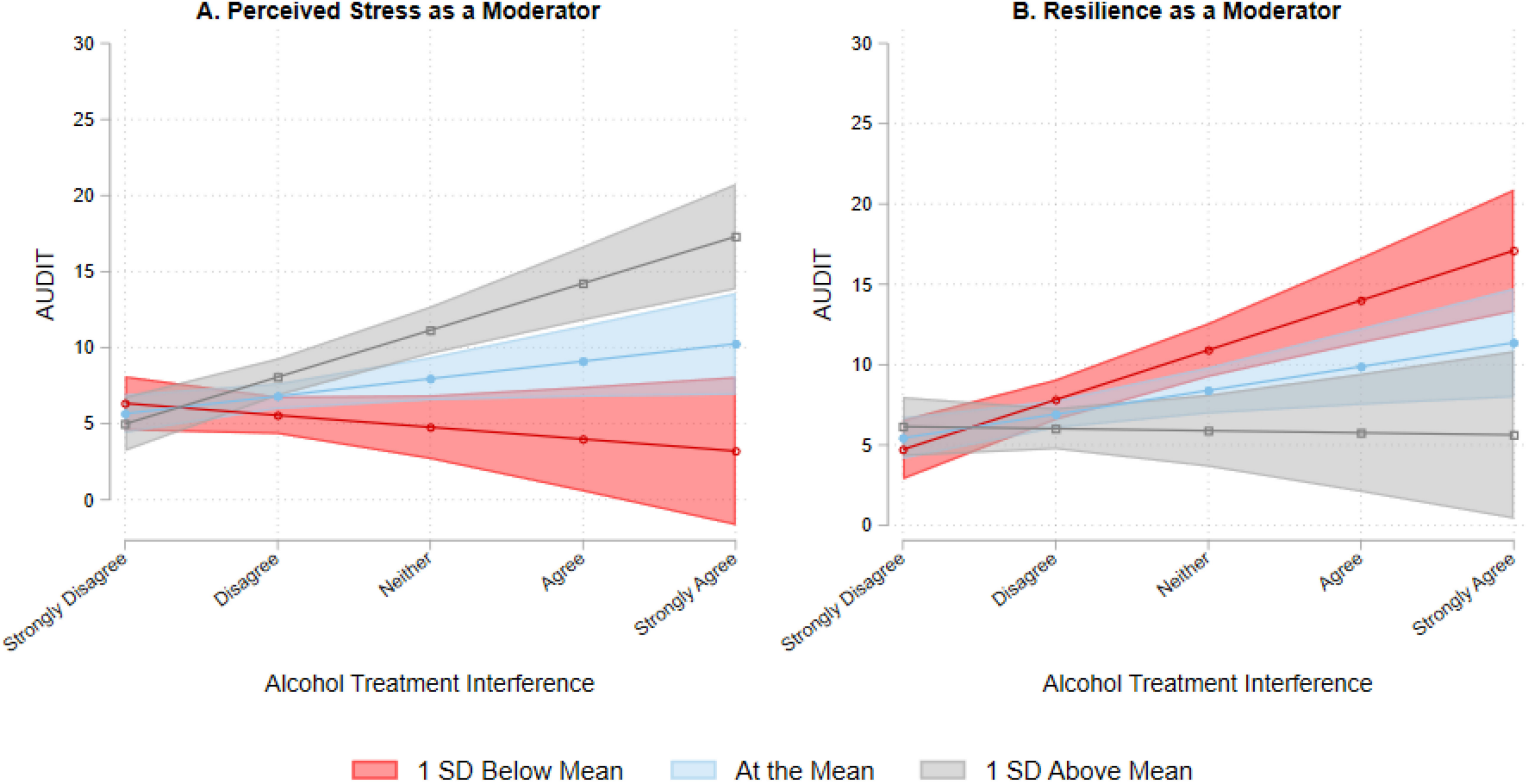
The association between alcohol treatment interference and problematic alcohol use with perceived stress **(A)** or resilience **(B)** as a moderator. AUDIT, Alcohol Use Disorders Identification Test.

Unadjusted and adjusted estimates from the multiple regression models are presented in **Table 2**. Significant interactions are visualized in **Figure 2**. Model 1 showed that AUD treatment interference was positively associated with higher problematic alcohol use (*b* = 2.05, *SE* = 0.53, *p* < 0.001). Model 2 indicated that perceived stress moderated the association between AUD treatment interference and problematic alcohol use (*b* = 0.24, *SE =* 0.05, *p* < 0.001). Simple slope analyses showed that the association between AUD treatment interference and problematic alcohol use was not significant at a low level (1 *SD* below the mean) of perceived stress (*b* = -0.78, *SE* = 0.77, *p* = 0.308), but was significant at the mean (*b* = 1.15, *SE* = 0.53, *p* = 0.032) and a high level (1 *SD* above the mean) of perceived stress (*b* = 3.08, *SE* = 0.58, *p* < 0.001). Model 3 showed that resilience also moderated the association between AUD treatment interference and problematic alcohol use (*b* = -0.09, *SE* = 0.03, *p* = 0.001). Simple slope analyses showed that the association between AUD treatment interference and problematic alcohol use was significant at a low level of resilience (*b* = 3.09, *SE* = 0.63, *p* < 0.001) and at the mean of resilience (*b* = 1.48, *SE* = 0.54, *p* = 0.007). The association between AUD treatment interference and problematic alcohol use was not significant at a high level of resilience (*b* = -0.13, *SE* = 0.82, *p* = 0.874). The main effect of AUD treatment interference and moderated effects of perceived stress and resilience were replicated in the sensitivity analysis among current drinkers only.

**Table 2:** Unadjusted and adjusted estimates from the multiple regression models on problematic alcohol use (N = 288).

	Model 1: Main Effect of Treatment Interference on Problematic Alcohol Use					
		Unadjusted			Adjusted	
	*b*	(95% CI)	*p*	*b*	(95% CI)	*p*
Treatment Interference	3.73	(2.58, 4.87)	<0.001	2.05	(1.001, 3.09)	<0.001
	Model 2: Interaction Effect of Treatment Interference and Perceived Stress					
	Unadjusted			Adjusted		
	*b*	(95% CI)	*p*	*b*	(95% CI)	*p*
Treatment Interference	-1.54	(-3.90, 0.81)	0.199	-2.32	(-4.37, -0.27)	0.027
Perceived Stress	-0.32	(-0.60, -0.04)	0.023	-0.32	(-0.56, -0.08)	0.009
Perceived Stress x Treatment Interference	0.28	(0.16, 0.40)	<0.001	0.24	(0.14, 0.34)	<0.001
	Model 3: Interaction Effect of Treatment Interference and Resilience					
	Unadjusted			Adjusted		
	*b*	(95% CI)	*p*	*b*	(95% CI)	*p*
Treatment Interference	9.94	(5.74, 14.14)	<0.001	7.74	(4.12, 11.35)	<0.001
Resilience	0.12	(-0.02, 0.27)	0.080	0.13	(0.01, 0.25)	0.032
Resilience x Treatment Interference	-0.10	(-0.17, -0.04)	0.002	-0.09	(-0.15, -0.04)	0.001

In the adjusted analyses, age, sex, race, ethnicity, years of education, household income, marital status, study enrollment phase, and history of AUD were included as covariates.

## Discussion

Parallel to research documenting how mental health treatment interference during the pandemic was associated with heightened psychological distress (27), the present research showed that AUD treatment interference was associated with higher problematic alcohol use. This is an important finding in light of recent research illustrating increased alcohol-related deaths in the United States during the pandemic (28). Possibly, interruption to AUD treatment could have contributed to increased alcohol-related morbidities (29), alcohol withdrawal (30), and alcohol use among individuals with AUD during the pandemic (31–33). The significant main effect of AUD treatment interference on problematic alcohol use supported the existence of an AUD treatment gap during the COVID-19 pandemic.

In this study, AUD treatment interference was assessed using a self-report item that gauged the subjective perception of whether individuals encountered disruption to treatment. To maximize generalizability of findings, we analyzed data from all participants who answered the question regardless of their prior AUD status as new problematic drinking behaviors could have emerged during the pandemic. While our sensitivity analysis ensured that the study findings were robust among current drinkers, replication and extension of our novel findings should be carried out in other clinical samples. Moreover, as telemedicine has become more widely adopted, research is needed to evaluate what type of addiction treatment may be effectively delivered online and identify ways to foster a sense of community using the online format. For some individuals, a return to in-person treatment sessions may be needed to optimize their AUD treatment experience.

Unique to the present investigation was the richness of the available data which allowed us to test perceived stress and resilience as moderators of the treatment interference and problematic alcohol use association. The significant interaction effects were consistent with our hypotheses: perceived stress strengthened the adverse impact of AUD treatment interference on problematic alcohol use and resilience exerted a protective effect. Clinical implications of this study include the need to foster positive coping strategies to mitigate the adverse impact of stress on drinking and to promote resilience as a buffer against problematic alcohol use (34, 35).

This study has several limitations. First, enrollment into the C19-PIA Study was on a rolling basis, and so variations in COVID-related restriction policies could have influenced the degree of AUD treatment interference. Second, a convenience sample was used and participants without income/education, resilience, or alcohol-related data were excluded. It is possible that participants with alcohol-related problems were less likely complete all measures which may introduce bias. To mitigate these concerns, future replications of the study findings in a larger clinical sample with a narrower study recruitment time frame and greater incentive for completing all study measures may be considered. Third, the AUD treatment interference construct was assessed using a single self-reported item and endorsement of treatment interference was low. This single item utilized a Likert scale and its use in regression analyses assumed that the intervals between response categories were equal. This measurement weakness can be addressed in future research with the use of more objective measures on treatment history, treatment type, and estimation of changes in the number of treatment sessions attended. Fourth, measures of perceived stress, resilience, and problematic alcohol use were also self-reported and may be vulnerable to recall or report bias. Extending the current findings, more research is needed to understand individual preferences for specific forms of addiction treatment delivery methods, such as in-person, telehealth, and hybrid formats (10), as well as the longer-term clinical outcomes after pandemic-related treatment interferences have subsided.

In conclusion, results from this study suggest the existence of an AUD treatment gap during the COVID-19 pandemic with treatment interruption linked to higher problematic alcohol use. Findings also highlight the importance of considering the moderating roles of contextual (e.g., perceived stress) and individual (e.g., resilience) factors in the association between AUD treatment interference and problematic alcohol use. To improve preparedness for future public health crises, the prioritization of cognitive-behavioral skills to improve stress management and promote resilience may be warranted to prevent problematic alcohol use or relapse to alcohol use (19). More broadly, the utilization of brief, mass-delivered interventions and lay-provider service delivery may also be an integral part of a comprehensive mental health response to future public health emergencies (36). While the shift to telehealth addiction treatment may have helped with improving access and convenience for some patients (8), more research is needed to examine individual preferences for various forms of AUD treatment and track the outcomes of these treatments in the post-pandemic world.

## Data Availability

The datasets presented in this article are not readily available because of ethical concerns regarding patient privacy and original patient consent. Data may be made available by requests directly to the corresponding authors. Requests to access the datasets should be directed to Jeremy Luk, jeremy.luk@nih.gov and Vijay Ramchandani, vijayr@mail.nih.gov.
